# Doxorubicin-Wrapped Zinc Oxide Nanoclusters for the Therapy of Colorectal Adenocarcinoma

**DOI:** 10.3390/nano7110354

**Published:** 2017-10-28

**Authors:** Sungyun Kim, Song Yi Lee, Hyun-Jong Cho

**Affiliations:** College of Pharmacy, Kangwon National University, Chuncheon, Gangwon 24341, Korea; vinny326@naver.com (S.K.); heymush@kangwon.ac.kr (S.Y.L.)

**Keywords:** anticancer activity, colorectal cancer, doxorubicin, nanocluster, zinc oxide

## Abstract

Doxorubicin (DOX)-wrapped zinc oxide nanoclusters (ZnO NCs) were developed for the therapy of colorectal cancer. DOX was coated onto the agglomerates of ZnO nanoparticles using a facile coating process. DOX-ZnO NCs with a hydrodynamic size of 170 nm, narrow size distribution, and positive zeta potential were fabricated. The aggregated shape of developed DOX-ZnO NCs was observed by transmission electron microscopy (TEM) imaging. The result of Fourier-transform infrared (FT-IR) analysis suggested the interaction between DOX and ZnO in DOX-ZnO NCs. The existence of DOX in the outer surface of DOX-ZnO NCs was further identified by X-ray powder diffractometer (XRD) and X-ray photoelectron spectroscopy (XPS) analyses. Cellular uptake efficiency and antiproliferation efficacy of developed DOX-ZnO NCs were tested in Caco-2 (human colorectal adenocarcinoma) cells. The cellular accumulated amount of DOX-ZnO NCs was 3.19-fold higher than that of free DOX (*p* < 0.05). The DOX-ZnO NCs group also exhibited improved antiproliferation potentials, compared with the DOX and ZnO groups, in Caco-2 cells at 0.5 and 1 μg/mL DOX concentrations. All these findings imply that developed DOX-ZnO NCs can be efficient hybrid nanoformulations for the therapy of colorectal cancers.

## 1. Introduction

Various therapeutic approaches have been developed for the tumor-targeted delivery of anticancer agents [1,2,3]. Among them, nano-size systems have been widely investigated due to their efficient cargo delivery to the tumor region after intravascular injection [4,5,6]. For fabricating nanocarriers, a lot of organic and inorganic materials have been used to provide tumor targetability [7,8]. The introduction of a specific ligand—which has a high binding affinity to the receptors expressed in cancers—into the nanocarriers can achieve active tumor targeting, as well as passive tumor targeting based on the enhanced permeability and retention (EPR) effect related to the physicochemical properties (i.e., particle size and surface charge) of nanosystems [9,10]. As organic matrices of nanocarriers, a couple of natural and synthetic polymers (i.e., hyaluronic acid, chitosan, chondroitin sulfate, poly(lactic-*co*-glycolic acid), and polyethylene glycol, etc.) have been investigated and their derivatives have also been synthesized to improve tumor targetability after intravenous injection [11,12,13,14,15,16]. Inorganic materials (i.e., Ag, Au, Ca, Cd, Cu, Fe, Gd, I, Mg, Mn, Si, Ti, and Zn) have been widely used for the imaging and therapy of various diseases [17,18,19,20]. These materials can be processed into nano-size systems with various shapes and multiple functionalities, such as nanocrystal, nanorod, nanoparticle, nanoshell, and nanowire [18,20]. Using the unique optical and electrical properties of those inorganic substances, there has been remarkable progress in the area of cancer diagnosis and therapy [18,20].

Among diverse inorganic substances, zinc has been widely used as a dietary supplement. It is known to be effective for the treatment of major depressive disorder, diarrhea, age-related macular degeneration, gastroenteritis, common cold, and sunburn. Zinc oxide (ZnO), as a source of zinc, exhibits unique catalytic, optical, piezoelectric, and semiconducting properties [21]. ZnO is classified as a “GRAS” (generally recognized as safe) material by the U.S. Food and Drug Administration [22]. However, its particle size change from micro to nano range needs further investigation regarding its toxicity [22]. In particular, ZnO can be dissolved in both acidic and strong basic conditions [21,23]. The outer surface of ZnO can be coated by various materials, such as small organic chemicals and inorganic materials, to expand its biomedical applications. In this investigation, doxorubicin (DOX), as a chemotherapeutic drug, was coated onto the outer layer of ZnO nanoparticles. ZnO nanoparticles are known to have cytotoxicities mainly based on the generation of reactive oxygen species (ROS) [22]. If the generated amount of ROS exceeds the cellular antioxidative capacity, it may lead to the cell death [22,24]. In this investigation, the anticancer effects of surface-modified ZnO nanoparticles were demonstrated in colorectal cancer cells. The Caco-2 (human colorectal adenocarcinoma) cell line was selected as a cell culture model of colorectal cancers. The anticancer activities of ZnO in Caco-2 cells were already revealed in previous studies [25,26]. Nano-sized ZnO particles may have higher toxicities than micro-sized ZnO particles due to the higher specific surface area [22]. However, the intravascular (i.e., intravenous) administration of micro-sized ZnO particles may be nearly impossible due to the unwanted side effects (i.e., embolization). In addition, nano-sized ZnO particles may have a passive tumor targetability mainly based on the EPR effect [9,10]. To amplify the anticancer activities of ZnO nanoparticles, DOX was attached to the outer layer of ZnO particles in this investigation. In the aqueous milieu, due to the high surface energy of ZnO nanoparticles, they are prone to be aggregated and hardly separated individually [27,28,29].

Herein, ZnO nanoclusters (NCs), as an agglomerated form of ZnO nanoparticles, were used instead of individual ZnO particles. DOX was then coated onto ZnO NCs to fabricate DOX-ZnO NCs. Their particle characteristics were systemically investigated and anticancer activities against Caco-2 cells were demonstrated in this study.

## 2. Materials and Methods

### 2.1. Materials

DOX (DOX HCl) was purchased from Boryung Pharmaceutical Co., Ltd. (Seoul, Korea). ZnO (nanopowder, 99.95% purity) was obtained from US Research Nanomaterials, Inc. (Houston, TX, USA). 2′,7′-Dichlorofluorescin diacetate (DCFH-DA) and propidium iodide (PI) were purchased from Sigma-Aldrich Co. (St. Louis, MO, USA). Dulbecco’s modified Eagle’s medium (DMEM), non-essential amino acids, penicillin, streptomycin, heat-inactivated fetal bovine serum (FBS), and phosphate buffered saline (PBS) were acquired from Gibco Life Technologies, Inc. (Grand Island, NY, USA). All other chemicals were of analytical grade and they were used without further purification.

### 2.2. Fabrication and Particle Characterizations of DOX-ZnO NCs

DOX-ZnO NCs were prepared by coating DOX onto the outer surface of ZnO nanoparticles. A ZnO nanopowder dispersion (5 mg/mL) in distilled water (DW) and a DOX solution (5 mg/mL) in DW were mixed at a 1:1 volume ratio and incubated for 1 h at room temperature. After centrifuging at 8000 rpm for 15 min, the supernatant was discarded, and the NC pellet was resuspended in DW. It was freeze-dried for further uses.

The hydrodynamic size, polydispersity, and zeta potential values of ZnO NCs and DOX-ZnO NCs were measured using dynamic light scattering (DLS) and laser Doppler methods (ELS-Z1000; Otsuka Electronics, Tokyo, Japan) according to the manufacturer’s instructions. ZnO NCs and DOX-ZnO NCs (at 5 mg/mL) were dispersed in DW using a probe-type sonicator (VC-750; Sonics & Materials, Inc., Newtown, CT, USA). The stability of ZnO NCs and DOX-ZnO NCs in serum media was also investigated using the DLS method. The NC dispersion and FBS were mixed at a 1:1 volume ratio and incubated for 24 h. The hydrodynamic size of the NC dispersion in serum medium was measured using the DLS method.

The content of DOX in DOX-ZnO NCs was quantitatively determined by fluorescence detection of DOX. Lyophilized DOX-ZnO NCs were dispersed in DW and the fluorescence intensity was detected at 480 nm (excitation wavelength) and 560 nm (emission wavelength) using an EMax Precision Microplate Reader (Molecular Devices, Sunnyvale, CA, USA). The standard curve of the DOX solution was used to calculate the content of DOX in DOX-ZnO NCs. The content of ZnO in DOX-ZnO NCs was quantitatively determined by inductively coupled plasma-optical emission spectrometry (ICP-OES; Optima 7300 DV, PerkinElmer, Inc., Waltham, MA, USA). DOX-ZnO NCs were dissolved in nitric acid with heating prior to its ICP-OES analysis.

The morphological shapes of ZnO NCs or DOX-ZnO NCs were observed by transmission electron microscopy (TEM) (JEM 1010; JEOL, Tokyo, Japan). ZnO NCs and DOX-ZnO NCs were dispersed at 5 mg/mL in DW by probe sonication (VC-750; Sonics & Materials, Inc.). An aliquot of NC dispersion was loaded onto a copper grid coated with carbon film and dried at room temperature. The mean diameter of individual nanoparticles was directly read from TEM images.

The released amounts of DOX from DOX-ZnO NCs were tested at pH 5.5. The aliquot (0.15 mL) of the dispersion of DOX-ZnO NCs (corresponding to 50 µg DOX) was put into the dialysis tube with a 14 kDa molecular weight cut-off (mini-GeBAflex tube with; Gene Bio-Application Ltd., Kfar Hanagide, Israel) [14,16]. The dialysis tube was loaded into 10 mL of release media (PBS, pH 5.5 adjusted with phosphoric acid) and then incubated in a shaking water bath (37 °C) at 50 rpm. Aliquots (0.2 mL) of the release media were collected at 6, 24, 48, 72, 96, 120, and 144 h, and replaced with an equivalent volume of fresh medium. The released amounts of DOX were determined by detecting the fluorescence intensity (EMax Precision Microplate Reader; Molecular Devices, Sunnyvale, CA, USA). Each sample was monitored at 480 nm (excitation) and 560 nm (emission) wavelengths.

### 2.3. Fourier-Transform Infrared (FT-IR) Analysis

FT-IR spectra of DOX, ZnO NCs, and DOX-ZnO NCs were acquired by using a Frontier™ FT-IR spectrometer (PerkinElmer Inc., Buckinghamshire, UK) with the attenuated total reflectance (ATR) mode. Transmittance (%) value of each sample was measured in the range of 400 to 4000 cm^−1^.

### 2.4. X-ray Powder Diffractometer (XRD) Analysis

XRD patterns of DOX, ZnO NCs, and DOX-ZnO NCs were obtained with a Philips X’Pert PRO MPD diffractometer (PANalytical Corp., Almero, The Netherlands) with a copper source (1.54 Å). Start and end angles (2θ) were set at 10° and 80°. The step size and time per step were 0.01° and 60 s, respectively. X-ray generator parameters were set as follows: 30 mA (current) and 40 kV (tension).

### 2.5. X-ray Photoelectron Spectroscopy (XPS) Analysis

The atomic compositions of ZnO NCs and DOX-ZnO NCs were measured by XPS (K-Alpha^+^, Thermo Fisher Scientific, East Grinstead, UK). The elemental percentages of Zn 2p, O 1s, N 1s, and C 1s in ZnO NCs and DOX-ZnO NCs were determined. The spot size and energy step size were 400 µm and 1 eV, respectively.

### 2.6. Cellular Uptake Studies

Caco-2 (human colon adenocarcinoma) cells were purchased from the Korean Cell Line Bank (Seoul, Korea). They were cultured with DMEM containing 10% FBS, 1% non-essential amino acids, 100 U/mL penicillin, and 0.1 mg/mL streptomycin at 37 °C in the atmosphere of 5% CO_2_ and 95% relative humidity. The cellular accumulation efficiency and intracellular distribution of DOX and DOX-ZnO NCs in Caco-2 cells were tested by flow cytometry and confocal laser scanning microscopy (CLSM), respectively. The fluorescence signals of DOX were used in the cellular uptake studies.

Caco-2 cells, at a density of 6.0 × 10^5^ cells per well, were seeded onto 6-well plates and incubated for 1 day at 37 °C. DOX solution or DOX-ZnO NCs dispersion, containing 10 μg/mL DOX, was added to the cells and they were incubated for 2 h. After eliminating treated samples, cells were then washed with PBS (pH 7.4) at least thrice. Cell pellets were collected by centrifugation and cells were suspended with PBS containing FBS (2%, *v*/*v*). Cell count according to the fluorescence intensity in each group was measured using a FACSCalibur fluorescence-activated cell sorter (FACS™) equipped with CellQuest software (Becton Dickinson Biosciences, San Jose, CA, USA).

The intracellular location of DOX solution and DOX-ZnO NCs in Caco-2 cells was observed by CLSM imaging. Caco-2 cells were seeded onto cell culture slides (1.7 cm^2^ surface area per well; BD Falcon, Bedford, MA, USA) at a density of 1.0 × 10^5^ cells per well and incubated for 1 day at 37 °C. DOX solution or DOX-ZnO NCs dispersion, containing 10 μg/mL DOX, was added to the cells and they were incubated for 2 h. Each sample was removed, and those cells were washed with PBS (pH 7.4) at least thrice. Then, they were fixed in 4% (*v*/*v*) formaldehyde solution for 10 min. They were dried under a gentle air stream, and VECTASHIELD mounting medium, including 4′,6-diamidino-2-phenylindole (DAPI) (H-1200; Vector Laboratories, Inc., Burlingame, CA, USA), was loaded onto the cells to stain the cell nuclei and prevent fluorescence quenching. The intracellular fluorescence signals were observed by CLSM (LSM 780; Carl-Zeiss, Thornwood, NY, USA).

### 2.7. In Vitro Anticancer Activity Tests

In vitro antiproliferation efficacy of developed NCs was tested in Caco-2 cells. After accomplishing 70–80% confluency in the cell culture dish, Caco-2 cells, at 5.0 × 10^3^ cells per well, were seeded onto a 96-well plate and were incubated for 1 day at 37 °C. DOX solution, ZnO NCs dispersion, or DOX-ZnO NCs dispersion, corresponding to 0.05, 0.1, 0.25, 0.5, and 1 μg/mL DOX concentrations, was added to the Caco-2 cells, and the cells were incubated for 72 h at 37 °C. After removing sample dissolved or dispersed medium, CellTiter 96^®^ AQ_ueous_ One Solution Cell Proliferation Assay Reagent (Promega Corp., Fitchburg, WI, USA), containing a tetrazolium compound (3-(4,5-dimethylthiazol-2-yl)-5-(3-carboxymethoxyphenyl)-2-(4-sulfophenyl)-2H-tetrazolium, inner salt; MTS) and an electron coupling reagent (phenazine ethosulfate; PES), was used to treat the cells, which were then incubated at 37 °C according to the manufacturer’s manuals. The absorbance in each group was detected at 490 nm using an EMax Precision Microplate Reader (Molecular Devices, Sunnyvale, CA, USA). Cell viability was calculated by comparing with that of the control (no treatment) group.

ROS-generation assay of developed NCs was performed in Caco-2 cells. After obtaining 70–80% confluency in the cell culture dish, Caco-2 cells were seeded onto a 6-well plate at a density of 3.0 × 10^5^ cells per well and were incubated for 1 day at 37 °C. DOX solution, ZnO NCs dispersion, and DOX-ZnO NCs dispersion, corresponding to 1 μg/mL DOX concentration, was used to treat the cells, which were then incubated for 24 h. Each sample was removed, and the cells were washed three times with PBS (pH 7.4) before being collected by centrifugation. Cells were treated with DCFH-DA solution (20 μM in PBS) and incubated for 1 h. Then, the cells were centrifuged at 1000 *g* for 5 min, the supernatant was discarded, and the cell pellet was resuspended with PBS containing FBS (2%, *v*/*v*). The cell population percentage, according to the intensity of DCFH-DA and PI in each group, was measured using a FACSCalibur fluorescence-activated cell sorter (FACS™) equipped with CellQuest software (Becton Dickinson Biosciences, San Jose, CA, USA). The population percentage in the lower left (LL) panel of the control (no treatment) group was set as 100%, and that of the other group in the lower right (LR) panel was presented to compare ROS generation.

### 2.8. Statistical Analysis

Each experiment was performed at least thrice and the data are presented as the mean ± standard deviation (SD). Statistical analysis was conducted using a two-tailed *t*-test and analysis of variance (ANOVA).

## 3. Results and Discussion

### 3.1. Fabrication and Characterizations of DOX-ZnO NCs

**Figure 1:**
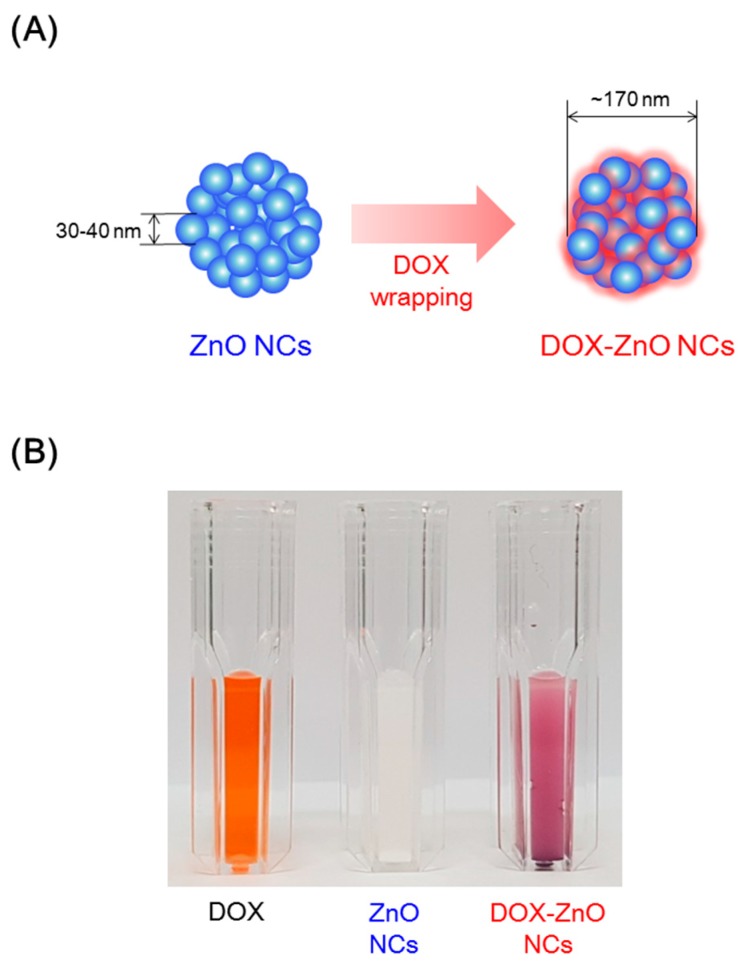
Development strategy of doxorubicin-wrapped zinc oxide nanoclusters (DOX-ZnO NCs). (**A**) Fabrication scheme of DOX-ZnO NCs; (**B**) Images of doxorubicin (DOX) solution, ZnO NCs dispersion, and DOX-ZnO NCs dispersion in distilled water (DW). DOX HCl, ZnO NCs, or DOX-ZnO NCs, corresponding to 10 mg/mL DOX-ZnO NCs concentration, were dissolved or dispersed in DW.

DOX-wrapped ZnO NCs were fabricated by a facile incubation protocol in this study. Although it is controversial whether or not free Zn^2+^ ions are a major contributor of cytotoxicity [22,30,31,32,33], ROS generation from ZnO has been regarded as the major cell-death mechanism [22]. To overcome the drawbacks of bare ZnO nanoparticles and improve their functionalities, the surface of ZnO nanoparticles has been engineered [27,29,34,35]. In this study, DOX (as a chemotherapeutic agent) was coated onto the outer surface of ZnO particles (Figure 1A). The different colors of DOX solution, ZnO NCs dispersion, and DOX-ZnO NCs dispersion, support the successful coating of ZnO NCs with DOX (Figure 1B). By combining DOX and ZnO into the single nanocarrier, the simultaneous arrival of DOX and ZnO in the tumor tissue and their efficient cellular entry may be possible.

**Figure 2:**
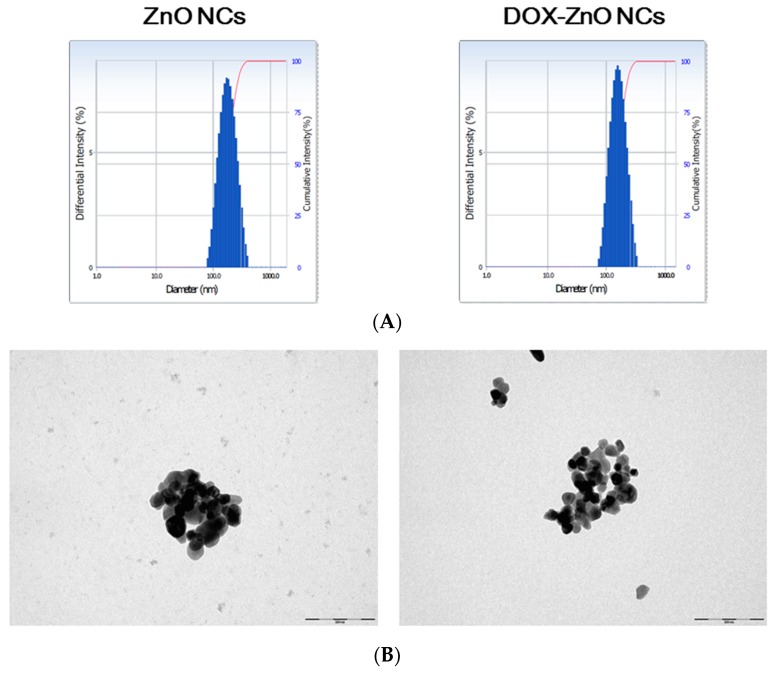
Particle characterizations of developed NCs. (**A**) Size distribution of dispersion of ZnO NCs and DOX-ZnO NCs. Differential intensity value according to the mean diameter of each formulation is plotted; (**B**) TEM image of dispersion of ZnO NCs and DOX-ZnO NCs. The length of the scale bar is 200 nm.

The mean diameter, read directly from TEM images, of ZnO nanoparticles was 44 ± 14 nm, and the hydrodynamic size (measured by DLS method) of ZnO NCs was 196 nm (Table 1 and Figure 2). This indicates that ZnO particles may exist as an aggregated form in the aqueous medium (e.g., water) (Figure 2B). Due to the aggregated form, they were named as a NC formulation in this study. The difference in particle size according to the measuring methods (i.e., individual particle size read from TEM image versus hydrodynamic size by DLS method) has already been reported elsewhere [36,37]. The average hydrodynamic size of DOX-ZnO NCs was 170 nm and the size distribution seemed to be narrow considering its polydispersity index (Table 1 and Figure 2A). Wrapping with DOX did not significantly affect the hydrodynamic size of ZnO NCs. As the zeta potential value of ZnO NCs was positive in this study, DOX coating did not alter the surface charge significantly. The average contents of DOX and ZnO in DOX-ZnO NCs were 1.32% and 97.43%, respectively. Considering the relatively low content of DOX compared to ZnO, DOX seems to be located in the outer layer of DOX-ZnO NCs.

The stability of developed NCs was tested in serum-included media (Figure S1). ZnO NCs exhibited a dramatic increment in the hydrodynamic size after mixing with serum media. On the contrary, the hydrodynamic size of DOX-ZnO NCs was significantly lower than that of ZnO NCs (*p* < 0.05). This implies that the DOX layer in DOX-ZnO NCs may contribute to the maintenance of colloidal stability even in serum-included media. The smaller hydrodynamic size of DOX-ZnO NCs, compared with ZnO NCs, in serum media may suggest more efficient cellular entry after intravenous administration.

The release pattern of DOX from DOX-ZnO NCs was tested at intracellular pH (pH 5.5) (Figure S2). The tested pH condition (pH 5.5) indicates the intracellular environment. The average released amounts of DOX from DOX-ZnO NCs after incubating for 6 h and 144 h were 29.0% and 92.8%, respectively. A sustained release pattern was observed, and almost the full amount of DOX was released from DOX-ZnO NCs after incubating for 6 days. This implies that DOX can be completely released from DOX-ZnO NCs after cellular entry and it may exert anticancer activities in cancer cells.

### 3.2. Solid-State Properties

**Figure 3:**
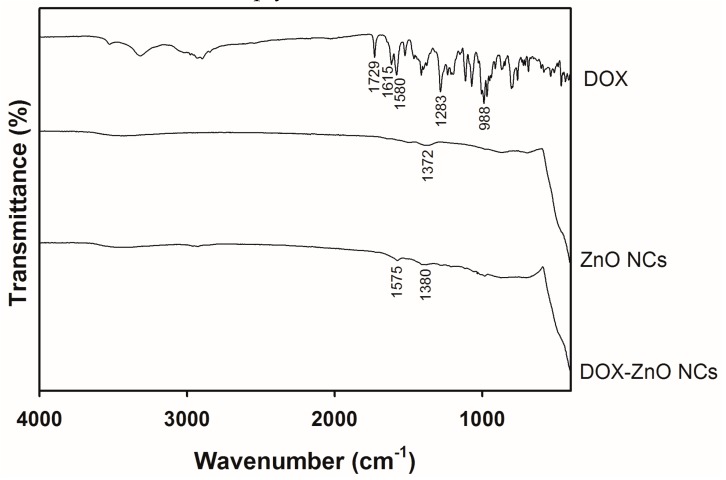
FT-IR spectra of DOX, ZnO NCs, and DOX-ZnO NCs. Transmittance according to the wavenumber of each sample is plotted.

**Figure 4:**
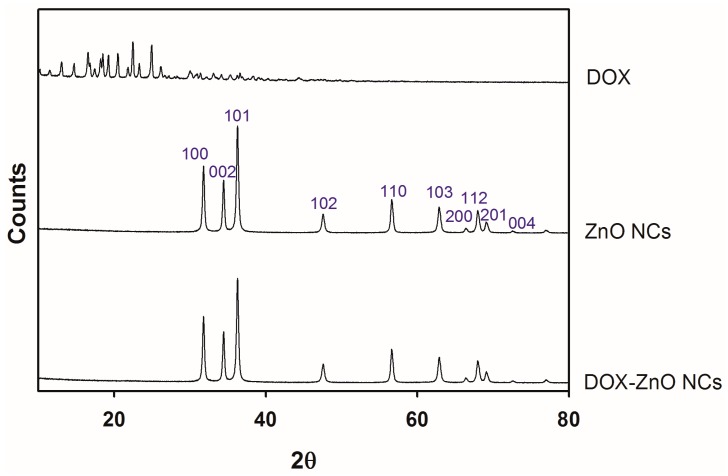
XRD profiles of DOX, ZnO NCs, and DOX-ZnO NCs. Counts value according to 2θ of each sample is plotted.

**Figure 5:**
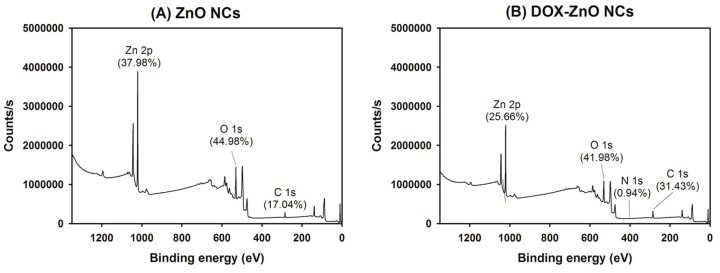
XPS results of (**A**) ZnO NCs and (**B**) DOX-ZnO NCs. Counts/s value according to binding energy of each sample is plotted.

The wrapping of ZnO NCs with DOX, and the interactions between ZnO and DOX, were investigated by solid-state studies (Figure 3, Figure 4 and Figure 5). The interactions between ZnO and DOX were studied by FT-IR analysis (Figure 3). Several peaks at 1729 (N–H), 1615 (CO–H), 1580 (aromatic C–H), 1283 (O–H···O, C–H, and C–OH), and 988 (C–C=O, C–OH, and aliphatic C–H) cm^−1^ were observed in the spectrum of DOX [38]. In the ZnO NCs group, a broad and weak peak was observed at around 3000 cm^−1^, indicating the hydroxyl group located on the surface of ZnO particles. Peaks at 400–500 cm^−1^ indicate the divalent metal oxide bond, as reported in [34,39]. After the coating of DOX onto the ZnO particles, the wavenumber and transmittance of peaks were altered as shown in the spectrum of DOX-ZnO NCs. The peak at 1580 cm^−1^ in the spectrum of DOX was shifted to 1575 cm^−1^ and its transmittance was alleviated in the spectrum of DOX-ZnO NCs. The peak at 1372 cm^−1^ in the spectrum of ZnO NCs was slightly shifted to 1380 cm^−1^ in the spectrum of DOX-ZnO NCs. All these FT-IR data imply the interactions between DOX and ZnO.

The thermodynamic state (crystalline versus amorphous) of developed NCs was investigated using XRD analysis (Figure 4). DOX exhibited multiple sharp peaks in the 2θ range of 10–40°, as reported in [40], indicating its crystallinity. In the ZnO NCs group, sharp diffraction peaks, corresponding to *hkl* values of 100, 002, 101, 102, 110, 103, 200, 112, 201, and 004 were shown at 2θ degree of 31.78°, 34.45°, 36.27°, 47.59°, 56.62°, 62.88°, 66.40°, 67.97°, 69.08°, and 72.55° [34,36]. This pattern means that ZnO has a polycrystalline wurtzite structure [36]. The XRD result of DOX-ZnO NCs exhibited a pattern more similar to that of ZnO NCs than that of DOX. Considering the content of DOX in DOX-ZnO NCs, the crystalline properties of ZnO seemed to be mainly shown in the DOX-ZnO NCs group.

The atomic compositions in the several outer layers of DOX-ZnO NCs were compared with those of ZnO NCs by XPS analysis (Figure 5). In the profile of ZnO NCs, Zn 2p (37.98%) and O 1s (44.98%) were presented. The element compositions in DOX-ZnO NCs were changed as follows: Zn 2p (25.66%), O 1s (41.98%), and N 1s (0.94%). The percentage of N 1s in DOX-ZnO NCs seemed to originate from the DOX molecule. The observed XPS data suggest that DOX may be successfully coated onto the ZnO particles during the fabrication process of DOX-ZnO NCs.

### 3.3. Cellular Uptake and Distribution

**Figure 6:**
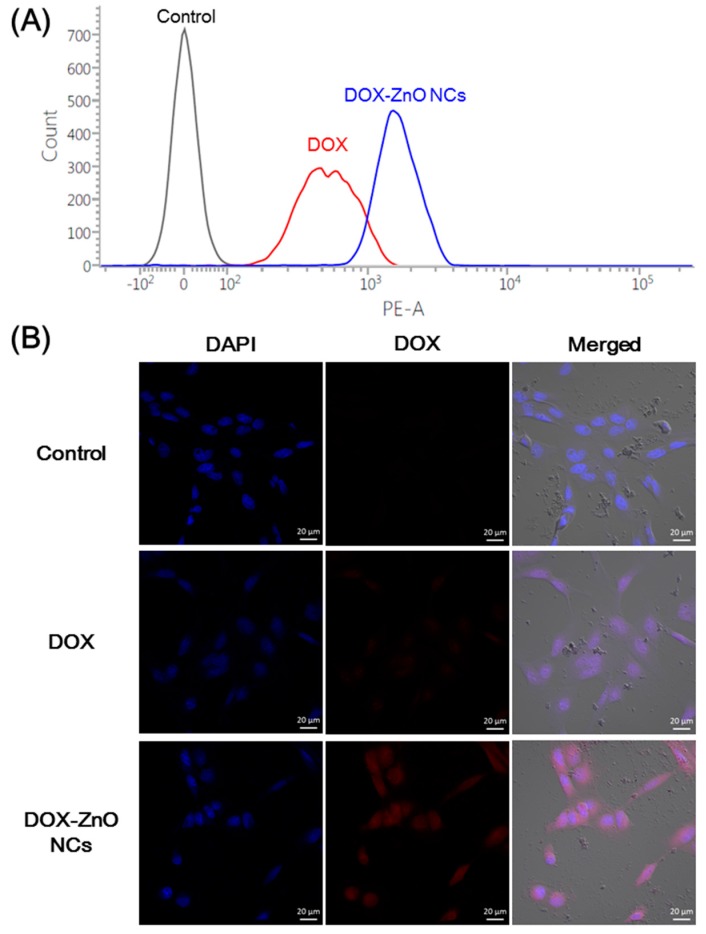
Cellular uptake tests of developed NCs in Caco-2 cells. (**A**) Cellular internalization efficiency of developed NCs in Caco-2 cells measured by flow cytometry. The cell count profiles according to the fluorescence intensity of each group after 2 h incubation are presented. Black, red, and blue colors indicate control, DOX, and DOX-ZnO NCs, respectively. Representative profile of triplicate is shown; (**B**) Intracellular distribution of NCs in Caco-2 cells observed by CLSM imaging. DOX and DOX-ZnO NCs were incubated for 2 h and CLSM images were acquired. Red and blue colors indicate DOX and 4′,6-diamidino-2-phenylindole (DAPI), respectively. The length of scale bar in the image is 20 μm.

The cellular accumulation and intracellular distribution of DOX-ZnO NCs were investigated by flow cytometry and CLSM analysis, respectively (Figure 6). The red fluorescence signal of DOX was used for evaluating the cellular uptake efficiency of fabricated DOX-ZnO NCs in this study. Caco-2 cells have been used as a model cell line of colorectal adenocarcinoma for assessing delivery systems of DOX [41]. Prior to cellular uptake experiments, the hydrodynamic size of the NCs in the cell culture media was measured using the DLS method. The mean diameter and polydispersity index of DOX-ZnO NCs in the cell culture media were 228 ± 16 nm and 0.12 ± 0.01, respectively. Considering those particle characteristics of DOX-ZnO NCs, efficient uptake of DOX-ZnO NCs into cancer cells may be expected. As shown in Figure 6A, the mean fluorescence intensity of the DOX-ZnO NCs group was 3.19-fold higher than that of the DOX group (*p* < 0.05). Such an increase in the cellular uptaken amount of the DOX-ZnO NCs group, compared with the DOX group, can be explained by the efficient cellular uptake of nanocarriers. The intracellular location of DOX and DOX-ZnO NCs was also compared by CLSM imaging (Figure 6B). Higher red fluorescence intensity was observed in the DOX-ZnO NCs group, compared with the DOX group. Of note, while DOX seemed to be diffused into the cells and located mainly in the nucleus, DOX-ZnO NCs were distributed in the cytoplasm as well as the nucleus. The higher fluorescence signal of the DOX-ZnO NCs group may indicate its efficient cellular accumulation in Caco-2 cells compared with the free DOX group.

### 3.4. In Vitro Anticancer Activities

**Figure 7:**
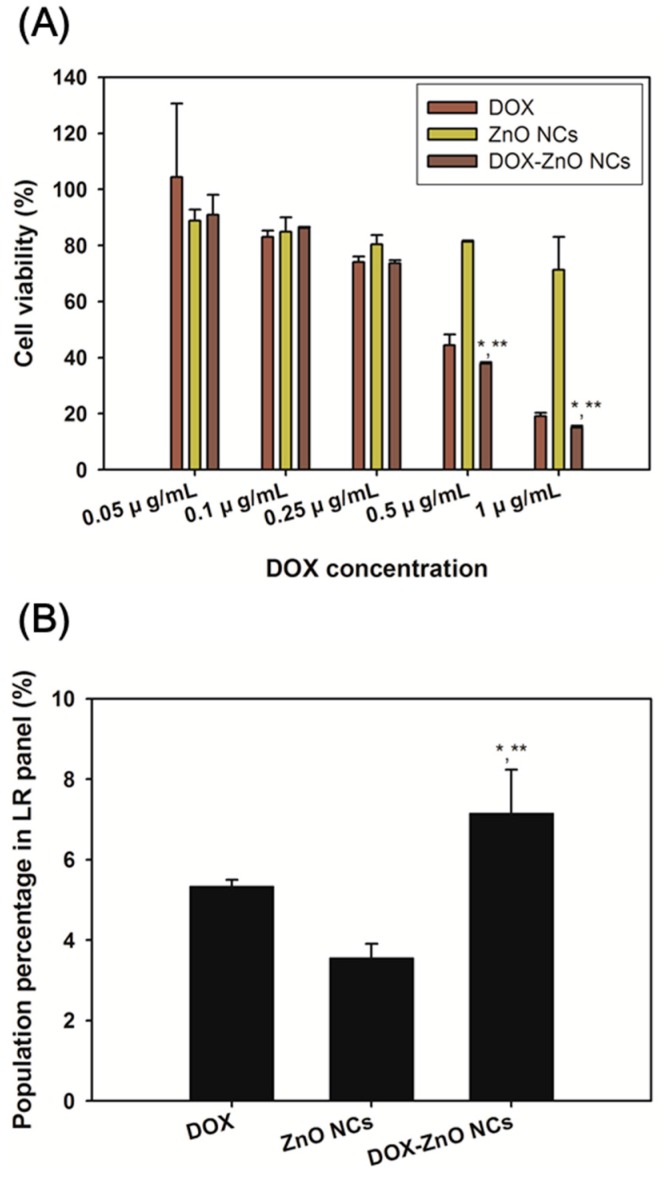
Anticancer activities of DOX-ZnO NCs in Caco-2 cells. (**A**) Antiproliferation efficacy of DOX-ZnO NCs in Caco-2 cells. Cell viability values of DOX, ZnO NCs, and DOX-ZnO NCs groups in Caco-2 cells are shown. Each point indicates the mean ± SD (*n* = 3). * *p* < 0.05, compared with DOX group. ** *p* < 0.05, compared with ZnO NCs group; (**B**) Reactive oxygen species (ROS) levels after treatment of DOX, ZnO NCs, and DOX-ZnO NCs in Caco-2 cells. The population percentages in the lower right (LR) panel of DOX, ZnO NCs, and DOX-ZnO NCs groups are shown. Each point indicates the mean ± SD (*n* = 3). * *p* < 0.05, compared with DOX group. ** *p* < 0.05, compared with ZnO NCs group.

In vitro antiproliferation activity of developed NCs was tested in Caco-2 cells (Figure 7A). MTS-based colorimetric assay was used to determine the number of viable cells. In metabolically active cells, the MTS tetrazolium compound (with the aid of PES) may be reduced to a colored formazan by reduced form of nicotinamide adenine dinucleotide phosphate (NADPH) or reduced form of nicotinamide adenine dinucleotide (NADH). The quantity of formazan produced was determined by measuring the absorbance at 490 nm, and may be proportional to the number of living cells. DOX, which has been used as a chemotherapeutic agent, exhibited antiproliferation activities in Caco-2 cells [42]. ZnO nanoparticles also showed cytotoxicity against Caco-2 cells through cellular oxidative stress [25,43]. The cytotoxicity of each component (i.e., DOX and ZnO) may guarantee the improved antiproliferation potentials of DOX-ZnO NCs. As shown in Figure 7A, there was no significant difference in cell viability of all experimental groups at relatively low DOX concentrations (0.05 and 0.1 μg/mL). However, at relatively high DOX concentrations (0.5 and 1 μg/mL), the DOX-ZnO NCs group exhibited significantly lower cell viability than the DOX and ZnO NCs groups (*p* < 0.05). Co-delivery of DOX and ZnO by using developed nanocarriers seemed to improve in vitro antiproliferation efficacies compared with the single treatment of DOX or ZnO NCs.

The up-regulation of intracellular ROS can trigger oxidative stress and, further, may lead to apoptosis [44]. It has already been reported that ZnO nanoparticles can induce apoptosis in human cancer cells via the production of ROS [36,45]. To elucidate the mechanism of improved antiproliferation efficacy of DOX-ZnO NCs (Figure 7A), ROS-generation assay was performed in Caco-2 cells (Figure 7B and Figure S3). Intracellular ROS levels were determined by detecting the population percentage of the LR panel (dichlorofluorescin (DCFH) positive and PI negative) [46]. After the cellular entry of DCFH-DA, it was deacetylated by cellular esterases and subsequently oxidized to DCFH by ROS. Therefore, the population percentage of the LR panel (DCFH positive and PI negative), determined by a flow cytometry, may imply the selective ROS level in living cells. The population percentage of the LR panel in the DOX-ZnO NCs group was significantly higher than those of the DOX and ZnO NCs groups (*p* < 0.05). The higher ROS level of the DOX-ZnO NCs group, compared with the DOX and ZnO NCs groups, may explain the improved antiproliferation efficacy in Caco-2 cells. The DOX group exhibited a higher ROS level compared with the ZnO NCs group, which may be due to their different cellular entry and cytotoxic action mechanisms. The antiproliferation potential of DOX was also higher than that of ZnO NCs as shown in Figure 7A. In addition, in the case of in vivo applications, each component (DOX or ZnO) may show different pharmacokinetic properties after intravenous administration. It is expected that the combination of DOX and ZnO into one carrier may amplify in vivo anticancer activities via simultaneous delivery of two substances in tumor tissues.

## 4. Conclusions

DOX-ZnO NCs were fabricated by a facile coating method. DOX-ZnO NCs with 170 nm hydrodynamic size, a narrow size distribution (0.09 polydispersity index), and positive zeta potential were successfully prepared. Individual ZnO nanoparticles seemed to be aggregated in aqueous media, and the DOX layer was coated onto the outer surface of the ZnO nanoparticles. DOX wrapping onto the ZnO NCs and the interactions between DOX and ZnO were investigated by solid-state studies (i.e., FT-IR, XRD, and XPS analyses). Cellular accumulation efficiency of DOX-ZnO NCs was 3.19-fold higher than that of DOX solution in Caco-2 cells. The DOX-ZnO NCs group exhibited improved antiproliferation potentials at 0.5 and 1 μg/mL DOX concentration, compared with the DOX and ZnO NCs groups, in Caco-2 cells. All these results indicate that developed DOX-ZnO NCs may be promising organic/inorganic hybrid nanoparticles for colorectal cancer therapy.

## Figures and Tables

**Table 1:** Particle characterizations of NCs (*n* = 3).

Composition	Hydrodynamic Size (nm)	Polydispersity Index	Zeta Potential (mV)
ZnO NCs	196 ± 18	0.12 ± 0.02	28.2 ± 3.3
DOX-ZnO NCs	170 ± 11	0.09 ± 0.03	27.8 ± 3.5

## Supplementary Materials

The following are available online at http://www.mdpi.com/2079-4991/7/11/354, Figure S1: Stability of NCs in serum media, Figure S2: Release profile of DOX from DOX-ZnO NCs at pH 5.5, Figure S3: Measurement of ROS levels after treatment of DOX, ZnO NCs, and DOX-ZnO NCs in Caco-2 cells.
