# Supplementary material for: Doxorubicin-Wrapped Zinc Oxide Nanoclusters for the Therapy of Colorectal Adenocarcinoma
Source: Nanomaterials (Basel). 2017 Oct 28;7(11):354. doi: 10.3390/nano7110354 (PMC5707571; doi:10.3390/nano7110354)
Supplement: Supplementary file 1 [file nanomaterials-07-00354-s001.pdf]

Supporting Information

Doxorubicin-wrapped zinc oxide nanoclusters for the therapy of colorectal adenocarcinoma

Sungyun Kim[1], Song Yi Lee[1], Hyun-Jong Cho[*]

College of Pharmacy, Kangwon National University, Chuncheon, Gangwon 24341, Republic of Korea

[1]These authors equally contributed to this work.
[*]Corresponding author. Tel.: +82 33 250 6916; fax: +82 33 259 5631.
E-mail address: hjcho@kangwon.ac.kr (H.-J. Cho).

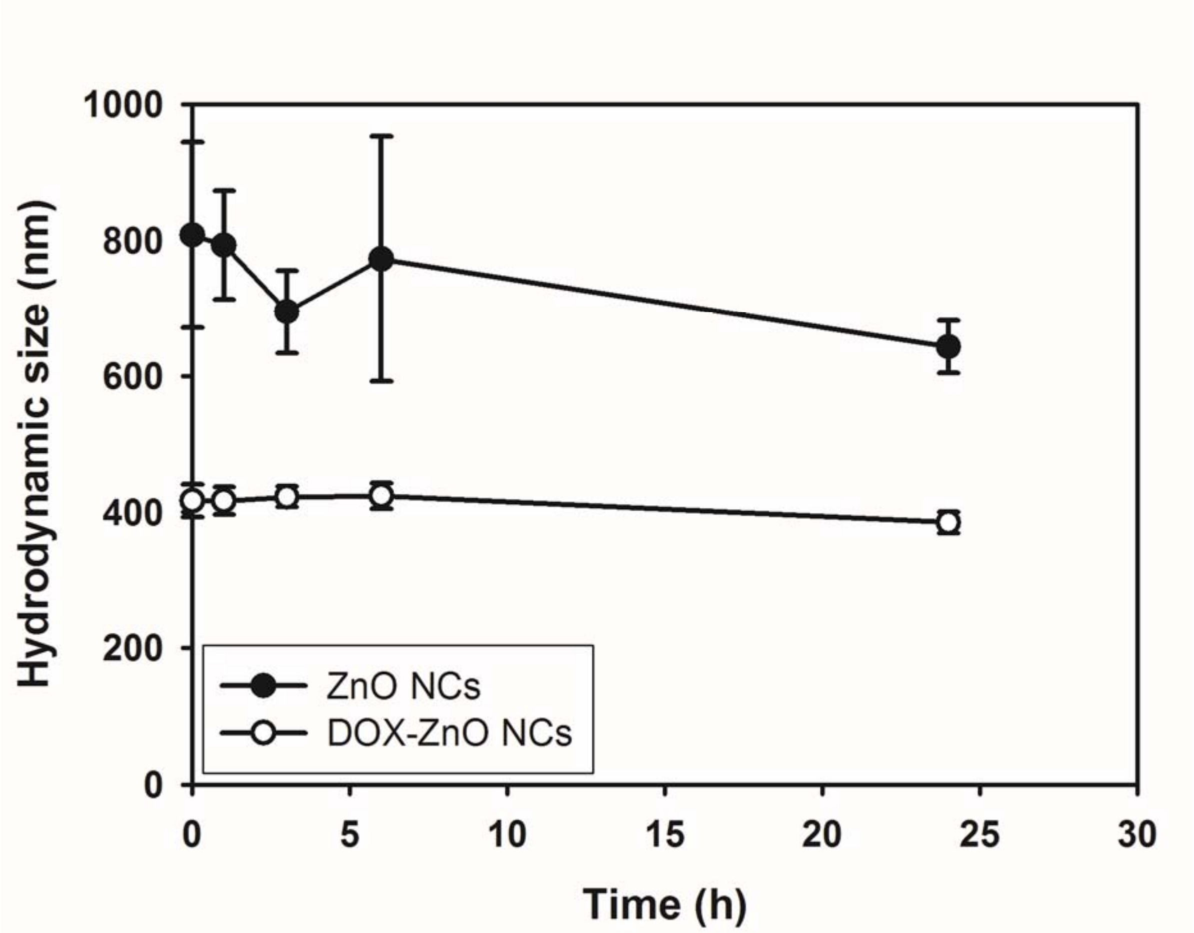

Figure S1. Stability of NCs in serum media. Hydradynamic size profiles of ZnO NCs and DOX-ZnO NCs, according to the incubation time, are presented. Each point indicates the mean ± SD (n = 3).

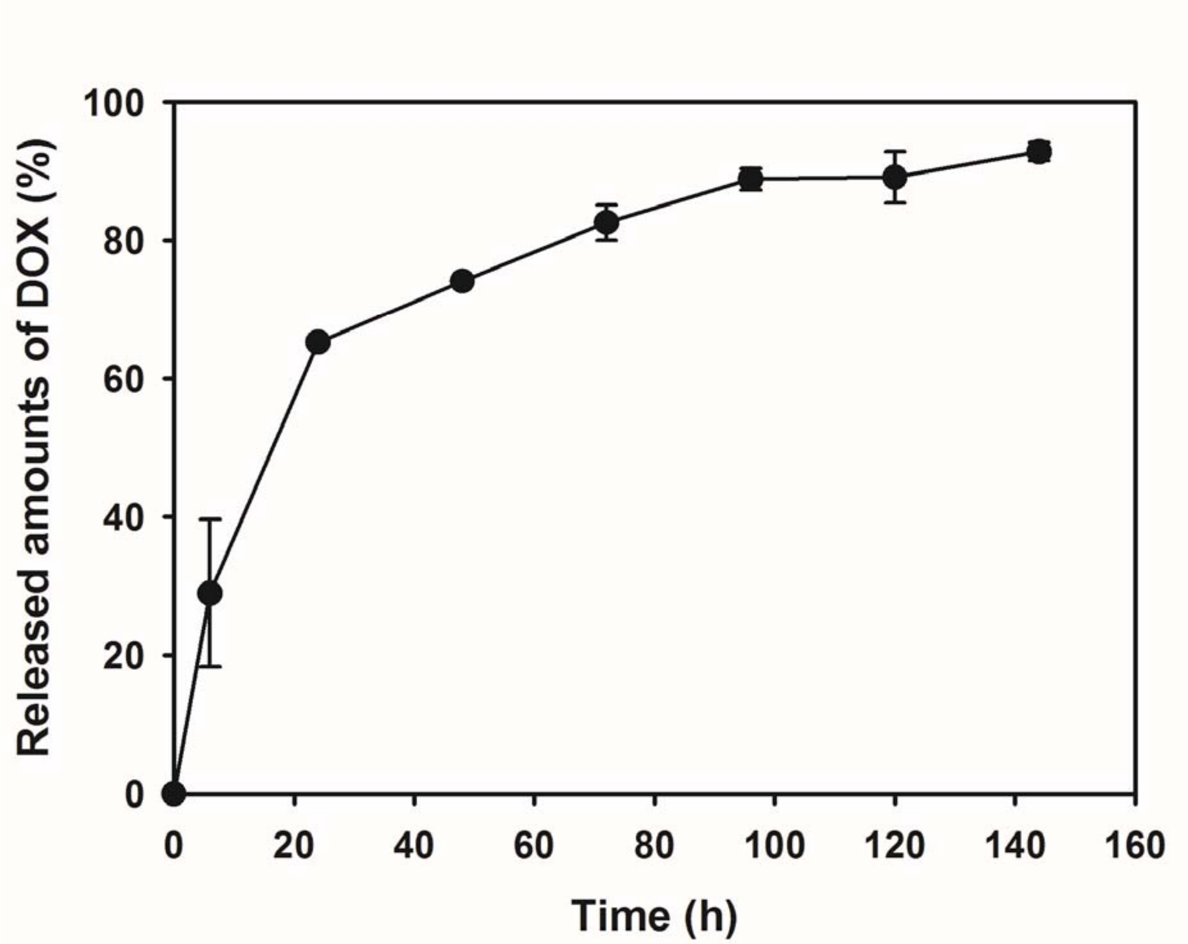

Figure S2. Release profile of DOX from DOX-ZnO NCs at pH 5.5. The released amounts (%) of DOX according to the incubation time are plotted. Each point indicates the mean ± SD (n = 3).

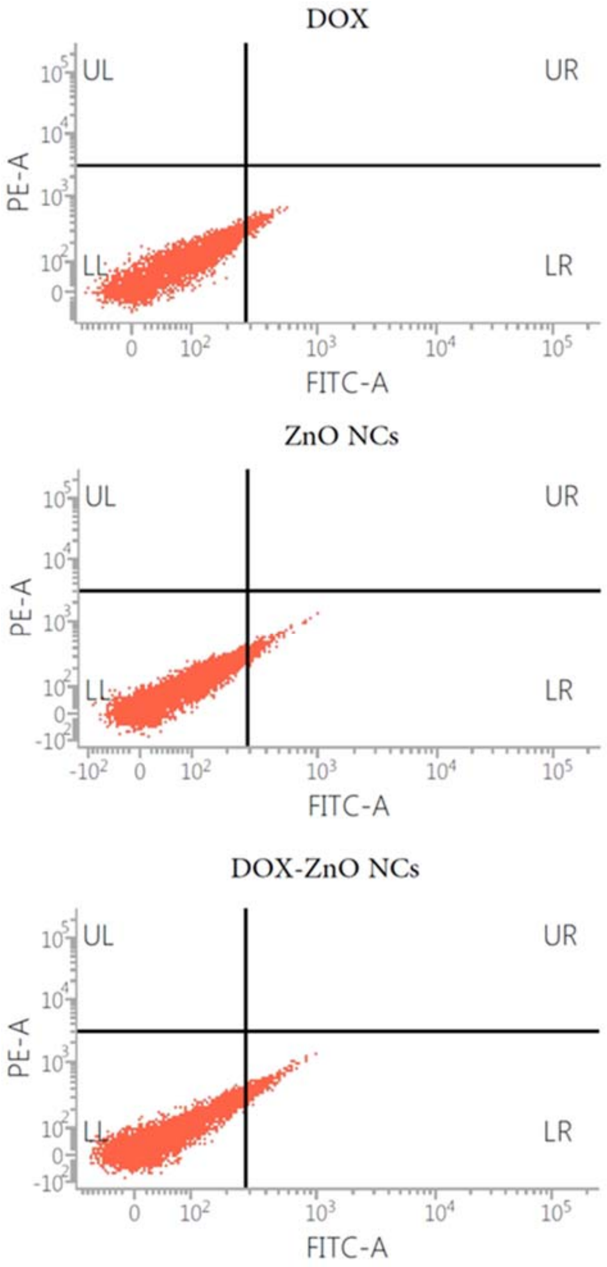

Figure S3. Measurement of ROS levels after treatment of DOX, ZnO NCs, and DOX-ZnO NCs in Caco-2 cells. Cell population diagrams, according to the intensity of DCFH (FITC-A) and PI (PE-A), of DOX, ZnO NCs, and DOX-ZnO NCs are presented. UL, LL, LR, and UR indicate upper left, lower left, lower right, and upper right, respectively.
